# Large Language Models in Bio-Ontology Research: A Review

**DOI:** 10.3390/bioengineering12111260

**Published:** 2025-11-18

**Authors:** Prashanti Manda

**Affiliations:** Department of Computer Science, University of Nebraska at Omaha, Omaha, NE 68182, USA; pmanda@nebraska.edu

**Keywords:** large language models, artificial intelligence, biological ontology, ontology mapping, knowledge graphs, semantic search, text mining, human-in-the-loop curation

## Abstract

Biomedical ontologies are critical for structuring domain knowledge and enabling integrative analyses in the life sciences. Traditional ontology development is labor-intensive, requiring extensive expert curation. Recent advances in artificial intelligence, particularly large language models (LLMs), have opened new possibilities to automate and enhance various aspects of bio-ontology research. This review article synthesizes findings from recent studies on LLM-assisted ontology creation, mapping, integration, and semantic search, while addressing challenges such as bias, reliability, and ethical concerns. We also discuss promising future directions and emerging trends that may further transform the way biomedical ontologies are developed, maintained, and used.

## 1. Introduction

Biomedical ontologies are formal, structured frameworks that provide standardized, computable representations of domain knowledge. They enable integrative analyses across diverse datasets by supporting data interoperability, semantic search, and computational reasoning. Foundational resources such as the Gene Ontology (GO) [1] and the Human Phenotype Ontology (HPO) capture essential biological and clinical concepts, while others—including the Disease Ontology (DO) [2], the Sequence Ontology (SO) [3], and ChEBI (Chemical Entities of Biological Interest) [4]—extend coverage to disease, genomic, and chemical domains. These ontologies are widely used in practice: in genomics and transcriptomics, GO annotations support functional enrichment analysis to uncover biological processes underlying experimental results [5], whereas in clinical medicine, HPO and DO facilitate patient phenotyping and differential diagnosis to advance personalized care [6]. Beyond these domain-specific applications, ontologies also underpin data mining and semantic search systems by enabling concept-based queries that go beyond simple keyword matching, thus enhancing both the precision and interpretability of biomedical knowledge discovery.

Despite their wide adoption, the creation and maintenance of biomedical ontologies remain highly challenging. Developing an ontology requires domain experts to define classes and relationships while ensuring consistency and completeness as new biological knowledge emerges [7]. This labor-intensive process has created a critical gap: as biomedical knowledge grows exponentially, traditional ontology engineering methods cannot keep pace with the demand for timely and comprehensive updates.

Historically, ontology construction has relied on large-scale, expert-driven collaborations. Foundational projects such as the Gene Ontology (GO) [1] and the Sequence Ontology (SO) [3] were built through years of iterative curation, consensus building, and peer review. These efforts demonstrated the value of structured semantic frameworks but also highlighted the substantial time and resources required, often involving extended community engagement over many years.

The movement toward community standards was further advanced by the OBO Foundry, which established principles for open and interoperable ontologies in the life sciences [8]. In parallel, large-scale clinical terminologies such as SNOMED CT [9] and integrative metathesauri like the Unified Medical Language System (UMLS) [10] extended ontology-based approaches into clinical and translational domains. While these initiatives broadened the impact of ontologies, they did not overcome the fundamental scalability problem: ontologies must be continuously updated to accommodate new discoveries, emerging diseases, and evolving clinical practices.

Consequently, ontology construction and curation remain time-consuming tasks, dependent on iterative manual review by domain experts [7,11]. With biomedical data expanding at an unprecedented pace, there is an urgent need for innovative approaches that can accelerate ontology engineering while maintaining quality and consistency. To alleviate this bottleneck, semi-automated text-mining and machine learning approaches have been explored, but they have generally required extensive feature engineering and still demanded significant expert validation [7,11]. With the exponential growth of biomedical literature and clinical data, these traditional methods have struggled to keep pace.

Recent advances in artificial intelligence, particularly large language models (LLMs) such as GPT-3 and GPT-4, have opened new possibilities for automating and enhancing bio-ontology research [12,13]. Trained on vast corpora, LLMs can understand complex biomedical language, extract semantic information from unstructured text, and generate human-like responses. In parallel, domain-adapted biomedical LMs such as BioBERT, SciBERT, PubMedBERT, and BioGPT provide stronger in-domain grounding that is directly relevant to ontology engineering tasks [14,15,16,17]. These capabilities make them promising tools for ontology engineering, where they can accelerate the generation of candidate terms and definitions, assist in mapping disparate ontologies, enrich text mining pipelines, and support interactive curation workflows [18,19]. Moreover, LLM outputs can be grounded to widely used biomedical standards and exchange schemas (e.g., HPO, GO, DO, MONDO, and the GA4GH Phenopacket schema) to support normalization and interoperability [20,21,22,23,24].

Unlike earlier rule-based or statistical NLP methods, LLMs capture subtle semantic relationships across biomedical and clinical text. Their ability to comprehend context allows them to suggest plausible ontology axioms, identify cross-ontology mappings, and facilitate ontology-guided semantic search. In this sense, LLMs represent a potential turning point for scaling ontology development, offering a level of flexibility and adaptability not feasible with previous approaches.

At the same time, significant challenges remain. LLM outputs are prone to factual errors and “hallucinations,” raising concerns about reliability in biomedical applications [25,26]. Models may also reproduce biases present in training data, which can propagate into ontology content if not carefully mitigated [27]. Furthermore, integrating probabilistic LLM outputs into the strict logical formalisms required by ontologies remains technically complex. Issues of interoperability, reproducibility, and the scalability of human-in-the-loop validation also pose persistent hurdles.

Together, these opportunities and challenges highlight the need for careful integration of LLMs into bio-ontology workflows. When paired with expert oversight, LLMs may enable faster, more responsive, and more comprehensive ontology development, but ensuring factual accuracy, ethical safeguards, and logical consistency will be critical for their success.

In this review, we examine how large language models (LLMs) may shift ontology engineering from a predominantly manual enterprise to a semi-automated, human-in-the-loop process, and how this transformation could reshape the pace and scope of biomedical knowledge integration. While LLMs are now widely applied across biomedical natural language processing (NLP), their specific role in ontology engineering has not been systematically explored. Prior surveys have summarized general LLM applications in bioinformatics [12], but none have provided a focused synthesis on ontology creation, curation, and integration. To address this gap, we organize our discussion into five thematic areas: (1) LLM-assisted ontology creation and curation, (2) ontology mapping and integration, (3) text mining and semantic search with ontologies, (4) automated ontology alignment, and (5) integration with knowledge graphs. Within each area, we highlight representative methods, emerging tools, and empirical findings, while also evaluating persistent challenges such as factual accuracy, bias, and reproducibility. By centering the analysis on ontologies, this review offers the first consolidated perspective on how LLMs can accelerate ontology development and maintenance, while identifying the obstacles that must be addressed before automation can be fully realized.

While large language models have become central to biomedical natural language processing—supporting applications such as entity recognition, relation extraction, and question answering—their systematic role in ontology engineering remains underexplored. Existing studies typically highlight isolated use cases, such as LLM-assisted text annotation or ontology term suggestion, without providing an integrated account of how these methods affect the ontology lifecycle as a whole. This review addresses that gap by synthesizing current evidence on how LLMs contribute to ontology creation, curation, mapping, and integration within the biomedical domain. By framing ontology engineering as a distinct application space for LLMs, we aim to establish a clearer understanding of both the opportunities and methodological challenges that arise when large language models are embedded in ontology-driven workflows.

## 2. Comparison to Prior Reviews

A number of recent surveys and position papers cover large language models (LLMs) in biomedicine, knowledge graphs (KGs), and ontology engineering [12,25,27,28,29,30,31,32,33]. However, their emphasis, scope, and methodological framing differ substantially from the present review.

This review fills a critical gap by providing the first bio-ontology-centric synthesis of large language model (LLM) applications across the full ontology lifecycle—from creation and curation to mapping and knowledge graph integration. Whereas prior reviews have primarily addressed general biomedical or clinical NLP use cases, this work explicitly focuses on ontology-centered tasks, offering a structured framework that connects LLM capabilities to the distinct stages, challenges, and governance needs of ontology engineering.

### 2.1. Scope and Audience

General surveys of LLMs in biomedicine primarily synthesize clinical and translational applications (e.g., QA, summarization, decision support), evaluation risks, and ethics [28,29,30]. These works are aimed at biomedical AI and clinical audiences and provide broad coverage of tasks and datasets. By contrast, our review targets bio-ontology engineering—curators, ontologists, and KG builders—focusing on how LLMs assist ontology creation, curation, mapping/alignment, and KG integration.

### 2.2. Ontology Engineering vs. Application-Centric Views

A recent systematic literature review on LLMs for ontology engineering synthesizes how LLMs assist core OE activities (requirements, implementation, publication, maintenance) and compiles task taxonomies and evaluation practices [31]. Our review complements that perspective by (i) centering on biomedical ontologies (e.g., OBO ecosystem) and (ii) integrating evidence from tools and pipelines that pair LLMs with curator workflows and logical/ID grounding (e.g., SPIRES/DRAGON-AI-style pipelines), with concrete implications for bio-curation communities and downstream biomedical reuse.

### 2.3. Alignment and Mapping: LLMs vs. Classical Matchers

Surveys and frameworks around LLMs for ontology matching (e.g., LLMs4OM) evaluate prompting and retrieval configurations for alignment, and position LLMs relative to classical systems [34]. Algorithmic papers (e.g., MILA) report state-of-the-art F1 on Ontology Alignment Evaluation Initiative (OAEI) biomedical tracks by combining heuristic search with selective LLM calls [35]. We synthesize these advances specifically through a bio-ontology lens—highlighting curator burden, precision/recall trade-offs for biomedical edge cases (rare terms, subtle phenotype distinctions), identifier normalization, and practical governance when promoting mappings into community ontologies.

### 2.4. LLMs, Knowledge Graphs, and Grounding

Recent reviews of healthcare knowledge graphs and position papers on unifying LLMs with KGs emphasize architectures, resources, and opportunities for neuro-symbolic integration [32,33]. Our treatment is narrower and more operational: we examine ontology-grounded generation and validation (e.g., KG-conditioned prompting, ontology-constrained relation extraction) and discuss how these patterns reduce hallucinations and improve interoperable identifiers in biomedical pipelines.

### 2.5. Risk, Evaluation, and Governance

Clinical/biomedical overviews articulate opportunities and risks (bias, privacy, factuality) and call for standardized evaluation in healthcare [28,36]. We translate these concerns to the ontology lifecycle: logical consistency, identifier fidelity, curator time-on-task, reproducibility of LLM outputs, and alignment with OBO/Findability, Accessibility, Interoperability, and Reuse (FAIR) governance. This review aggregates concrete mitigation strategies—ontology-aware prompting, retrieval for ID grounding, reasoner checks, and human-in-the-loop gates—tailored to ontology production.

### 2.6. Summary of Differentiators

Compared to prior reviews, our contributions are

(a) A bio-ontology-centric synthesis of LLM use across creation, curation, mapping/ alignment, and KG integration, rather than general biomedical applications;
(b) A methods-to-workflow bridge that surfaces where LLM suggestions enter curator pipelines and how ontology logic/IDs constrain outputs;
(c) A precision view of alignment in biomedical settings (phenotypes, diseases, anatomy), emphasizing curation cost and governance when adopting LLM-generated mappings;
(d) A grounding and reproducibility focus, consolidating practices (Retrieval Augmented Generation (RAG) to canonical IDs, reasoner checks, audit trails) that are specific to ontology/KG ecosystems.

### 2.7. Where This Review Adds Unique Value

This review adopts an ontology-first perspective, offering a structured account of how LLMs accelerate each stage of the ontology lifecycle (creation → mapping → KG integration → applications) while detailing ontology-specific risks and mitigations. We take a comparative lens on alignment by positioning LLM-augmented matchers alongside AML/LogMap-style classical systems and indicating where curator triage delivers the greatest return on effort [34,35]. Building on that, we distill actionable curation patterns—grounding via identifier normalization and ontology-constrained prompting, auditable traces, and human-in-the-loop acceptance—that operationalize LLM outputs for OBO workflows, a level of operational detail rarely covered in broader LLM-in-health surveys [28,30,36]. In contrast to general medical/biomedical LLM surveys [28,29,30] and KG-focused syntheses [32,33], our contribution centers on bio-ontology engineering as the organizing axis, providing fine-grained coverage of LLM-assisted creation, mapping/alignment, ontology-grounded extraction, and KG integration alongside guidance for curator-governed adoption. Compared with the dedicated SLR on LLMs for ontology engineering [31], we additionally integrate results from KG-grounded RAG and neuro-symbolic validation, connect them to OBO governance, and propose evaluation criteria tailored to ontology maintenance at scale.

It is important to note that this review focuses specifically on ontology-centered applications of large language models—covering ontology creation, curation, mapping, and integration—rather than on general biomedical or clinical NLP use cases such as diagnosis prediction, report summarization, or medical dialogue generation. By delimiting the discussion to ontology engineering tasks, we aim to provide depth and clarity on how LLMs interface with structured biomedical knowledge frameworks, rather than reiterating broader trends in biomedical AI.

## 3. Background

In this section, we synthesize key findings from the literature and categorize them into five thematic areas: (1) LLM-Assisted Ontology Creation and Curation, (2) LLMs for Ontology Mapping and Integration, (3) Text Mining and Semantic Search with Ontologies, and (4) Automated Ontology Mapping and Alignment, and (5) Knowledge Graphs and Ontology Integration.

Ontology engineering differs fundamentally from general natural language processing (NLP) tasks in both objective and structure. While general NLP focuses on understanding, generating, or classifying unstructured language, ontology engineering requires the formal representation of knowledge through logically consistent classes, relations, and axioms. This distinction means that integrating LLMs into ontology workflows involves additional constraints: outputs must adhere to strict logical formalisms, align with established identifiers, and remain interoperable with existing ontology frameworks. Unlike tasks such as summarization or named entity recognition, ontology construction demands precision, explicit semantics, and reproducibility—areas where the probabilistic nature of LLMs introduces unique challenges.

### 3.1. LLM-Assisted Ontology Creation and Curation

Traditional ontology development is heavily reliant on manual effort. Researchers have demonstrated that LLMs can be leveraged to generate candidate ontology axioms from natural language inputs [18]. In the SPIRES framework, for example, GPT-based models produce draft axioms that capture semantic relationships, which experts can then verify and refine. This approach not only reduces the time required for initial ontology population but also provides a dynamic means of updating ontologies as new literature emerges.

Building and updating ontologies traditionally demands substantial effort from domain experts and ontologists [19]. A major theme in recent work is using LLMs to semi-automate ontology creation and curation. Rather than replacing human expertise, LLMs serve as intelligent assistants to accelerate ontology development. For example, Joachimiak et al. (2024) describe the Artificial Intelligence Ontology (AIO)—an ontology of AI concepts—which was developed via manual curation with additional assistance from LLMs [37]. In their approach, the team leveraged large models to help with concept recognition and keep the ontology up-to-date with the rapidly evolving AI domain. The AIO’s content is dynamically updated through AI-driven curation support, ensuring the ontology remains relevant as new AI methodologies and terms emerge [37]. This demonstrates how LLMs can continuously suggest new terms or relations for curators to consider, thereby speeding up the evolution of an ontology in a fast-moving field (in this case, the AI domain, but the strategy is generalizable to biomedical domains).

Similarly, the DRAGON-AI pipeline introduced by Toro et al. [19] exemplifies the integration of retrieval-augmented generation (RAG) with large language models (LLMs) to enhance ontology development. By querying biomedical literature and existing ontologies, DRAGON-AI generates candidate classes, relationships, and definitions, significantly streamlining the ontology curation process. While human validation remains essential, this system effectively narrows the curator’s focus to the most promising candidate entries, expediting ontology construction.

DRAGON-AI has been evaluated across ten diverse ontologies, demonstrating high precision in generating hierarchical relationships between concepts. Notably, it was able to generate textual definitions for new terms, with expert evaluators finding them largely acceptable, albeit slightly lower in quality than human-written definitions. This underscores the importance of expert oversight, as domain specialists were better equipped to identify subtle inaccuracies in AI-generated content. Nonetheless, the ability of LLMs to draft initial ontology components provides a valuable starting point, reducing the manual effort required for ontology construction.

A particularly compelling feature of DRAGON-AI is its ability to incorporate natural language instructions, such as GitHub issue requests for new terms, into the ontology update process. This points to a future where interactive, human-in-the-loop workflows enable dynamic and responsive ontology engineering. These capabilities align with findings by Joachimiak et al. [37], who demonstrated that LLMs can assist in maintaining up-to-date ontologies, particularly in fast-evolving domains like Artificial Intelligence Ontology (AIO). Together, these efforts highlight the growing role of LLMs as intelligent assistants in ontology curation, where AI-generated drafts provide a foundation that domain experts refine, enabling a more scalable, efficient, and adaptive approach to ontology development.

Beyond SPIRES and DRAGON-AI, recent case studies have shown LLM-supported workflows for constructing specialized ontologies (e.g., clinical trial domains) and maintaining high-velocity concept spaces such as the AI Ontology, with curation pipelines integrated into community platforms like BioPortal [37,38].

Beyond fully automated generation, LLMs are being used as intelligent assistants for ontology editing and curation tasks. Kommineni et al. (2024) proposed an LLM-supported semi-automatic pipeline for ontology and knowledge graph construction [39]. Their approach starts with formulating competency questions (functional questions the ontology should answer), then uses those to guide ontology schema creation and instance population. An open-source LLM is employed to extract candidate ontology terms and facts from scholarly publications, building a knowledge graph that is then evaluated within the scope of the program.

To assess the quality of automatically generated content, they introduced a “judge LLM” that rates the generated triples against ground truth, simulating an expert review. In a case study on creating a knowledge graph of deep learning methods, this pipeline reduced the needed human effort while still producing a useful ontology and knowledge base. However, the authors note that a human-in-the-loop approach is still recommended to verify the LLM’s output before deployment. This reflects a common theme: LLMs can take over some of the grunt work (e.g., scanning text for relevant knowledge, drafting ontology entries), but human experts should remain in control of approval and integration of the content.

LLMs have also shown value in curating specific ontology content from literature. Mukanova et al. developed a method to convert natural language text into ontology instances using ChatGPT (GPT-4) [40]. In their use case, they took textual descriptions about geographic and administrative regions of Kazakhstan and used an LLM to extract entities and relationships aligned with an ontology of the region. The extracted information was then programmatically inserted as individuals and properties in the ontology (LLM-Powered Natural Language Text Processing for Ontology Enrichment). This automated text-to-ontology pipeline enriched the ontology with real-world facts (e.g., province names, borders, population data) that were previously only in unstructured documents. Notably, the approach was domain-agnostic—by changing the ontology and providing relevant texts, the same technique could be applied to biomedical domains for populating ontologies with instances from scientific literature or databases. The authors showed that such LLM-powered enrichment can significantly improve the efficiency of maintaining knowledge bases, essentially bridging unstructured natural language and structured ontological data (LLM-Powered Natural Language Text Processing for Ontology Enrichment). Similar ideas are being explored in biomedical contexts, for example using GPT-based tools to suggest new disease or gene entries for ontologies by reading journal articles.

Early results indicate that LLMs are adept at drafting definitions or finding relevant relationships, but careful curator review is needed to catch occasional mistakes or omissions. In summary, across ontology creation and curation, LLMs are proving to be valuable collaborators—accelerating the addition of new knowledge and reducing curator workload—while domain experts provide guidance and quality control. Efforts such as AIO and DRAGON-AI show that when LLMs are integrated into ontology workflows, the ontology can evolve more rapidly to capture emerging knowledge [19,37]. The paradigm emerging is one of human–AI co-creation: LLMs handle the bulk knowledge extraction and drafting, and humans handle the validation, corrections, and final decisions. This synergy can make ontology engineering more scalable and responsive to new information than ever before.

While early reports of LLM-assisted tools such as SPIRES [18] and DRAGON-AI [19] suggest they can accelerate ontology development, the extent of this improvement compared to traditional workflows remains an open question. Historically, ontology creation relied on expert committees manually drafting definitions and relationships, a process often spanning months or years for major resources such as GO or HPO [1,8]. LLM-based tools appear to reduce the upfront drafting burden by automatically proposing candidate terms, definitions, and mappings, but they also introduce a new layer of verification. For example, SPIRES showed that while curators spent less time generating definitions, they devoted significant effort to validating model outputs and correcting hallucinated content [18]. In practice, therefore, LLMs may shift the curator workload from content generation to content auditing, with net time savings depending on the domain, task complexity, and error rate of the model. A systematic, quantitative comparison of expert time spent on drafting versus reviewing remains largely absent, and developing such benchmarks will be essential for demonstrating the practical value of LLM-based ontology curation.

Despite their promise, practical deployments of LLM-assisted ontology tools have also revealed consistent failure modes that underscore the need for cautious integration. For instance, SPIRES and DRAGON-AI occasionally produce fabricated or non-resolvable identifiers (e.g., invalid GO or HPO IDs) when grounding confidence is low, requiring curators to manually verify and replace them. Logical inconsistencies are another recurring issue—generated axioms may violate domain or range restrictions, introduce circular class dependencies, or conflict with existing hierarchy constraints. Moreover, stylistic deviations from OBO Foundry principles are common: definitions sometimes use colloquial phrasing, omit necessary genus–differentia structure, or fail to reference authoritative sources. In practice, curators report that resolving these issues can consume as much effort as the initial generation step, emphasizing that LLMs are most effective when embedded within structured review pipelines that enforce identifier validation, reasoning checks, and editorial style compliance. Including these safeguards transforms LLMs from autonomous generators into dependable co-curation assistants aligned with OBO quality standards.

In summary, LLM-based ontology alignment systems such as MapperGPT and MILA demonstrate clear advantages in handling semantically complex or lexically distant mappings, often achieving higher recall by recognizing contextual equivalence that classical tools overlook. In contrast, traditional matchers like AML and LogMap typically deliver superior precision and logical consistency, benefiting from deterministic rules and built-in reasoning constraints. A practical hybrid workflow can therefore combine the strengths of both paradigms: high-confidence mappings are resolved using classical methods, while uncertain or ambiguous candidate pairs are escalated to an LLM for contextual verification. This tiered approach allows curators to capitalize on LLMs’ semantic flexibility without compromising the precision and reproducibility central to ontology integration, achieving an effective balance between automation and expert oversight. Overall, LLMs in ontology creation and curation are proving to be valuable collaborators, particularly for initial term suggestion and hierarchical structuring. However, the need for human oversight remains, as subtle domain-specific nuances often require expert judgment.

### 3.2. Ontology Mapping and Integration

Bio-ontologies rarely exist in isolation—integrating knowledge across them is essential for data interoperability. Ontology mapping, or matching, identifies correspondences between entities across different ontologies, enabling data annotated with one vocabulary to be compared or merged with another. The task remains difficult due to terminological variation, inconsistent hierarchies, and differences in granularity. Classical alignment systems such as AgreementMakerLight (AML) and LogMap rely on lexical, structural, and logical similarity measures [41,42,43,44]. These methods achieve strong precision and reproducibility but often miss semantically complex correspondences that require contextual interpretation.

Recent work demonstrates that coupling classical matchers with large language models (LLMs) can substantially improve semantic recall and curator efficiency [34,45]. Systems such as MapperGPT and MILA (Map with Iterative LLM-Assisted Search) exemplify this hybrid strategy. MapperGPT integrates high-recall lexical mapping with LLM-based semantic refinement: for each candidate pair, the model evaluates whether two entities represent the same concept based on their definitions and contextual descriptions [46]. Evaluations across anatomy, developmental biology, and renal disease ontologies showed that MapperGPT outperformed LogMap by achieving higher precision without reducing recall, accurately aligning lexically dissimilar but semantically equivalent concepts. By limiting LLM queries to ambiguous cases, MapperGPT acts as a “semantic curator,” balancing accuracy with computational efficiency.

MILA extends this principle through a prioritized depth-first search strategy that consults the LLM only for uncertain mappings [35]. Straightforward matches are resolved using fast lexical embeddings, while difficult or ambiguous ones are escalated to the LLM. This retrieve–identify–prompt pipeline achieved state-of-the-art results in the 2023–2024 OAEI biomedical alignment challenges, obtaining the highest F1 scores in four of five unsupervised matching tasks. MILA’s selective prompting substantially reduced LLM calls, demonstrating that combining heuristic search with targeted model reasoning can yield both efficiency and accuracy.

Additional hybrid frameworks reinforce this trend. Ruan et al. [47] proposed an LLM-driven refinement method that filters candidate mappings from traditional matchers, and Cavalleri et al. [48] demonstrated iterative prompting with human-in-the-loop review through SPIREX, improving mapping accuracy when lexical cues are insufficient. These studies highlight that LLMs can complement symbolic systems by injecting semantic flexibility while maintaining curator oversight.

Benchmarking efforts now explicitly evaluate such methods. The OAEI biomedical tracks [49] and LLM-focused initiatives (LLMs4OM, OAEI-LLM) provide standardized testbeds for measuring performance on complex mappings [34,50]. Standardized output formats such as Simple Standard for Sharing Ontology Mappings (SSSOM) ensure that LLM-generated correspondences remain transparent, auditable, and shareable [51]. Beyond static evaluation, practical criteria such as curator time saved and validated suggestion rate are increasingly being used to assess real-world impact.

Beyond mapping, LLMs are also being integrated with ontology reasoning and structure-aware learning. Failure analyses of identifier linking and coverage “deserts” have guided prompt and retrieval design for alignment workflows [52]. Neuro-symbolic frameworks such as RELATE enforce ontology constraints during or after generation, reducing spurious alignments and preserving logical consistency [53]. Ontology-enhanced contrastive tuning improves biomedical similarity judgments [54], ontology-constrained generation minimizes nonsensical relations in extraction tasks [55,56], and ontology-driven self-training frameworks such as OntoTune [57] align model representations to hierarchical biomedical ontologies while maintaining general linguistic competence. Systems like GenOM [58] further enrich sparse ontology labels with LLM-generated descriptions before matching, achieving superior recall on biomedical alignment tasks.

In summary, LLM-assisted ontology mapping systems such as MapperGPT, MILA, SPIREX, and OntoTune demonstrate that hybrid neuro-symbolic integration can deliver tangible gains in semantic accuracy, scalability, and curator productivity. LLMs enhance recall on semantically complex mappings, while classical systems retain superior precision and reproducibility. A practical workflow combines both paradigms: high-confidence mappings are resolved by deterministic algorithms, and ambiguous cases are reviewed through LLM reasoning and curator validation. As biomedical ontologies continue to grow in number and complexity, such adaptive hybrid pipelines will be essential for scalable, semantically robust ontology integration.

### 3.3. Text Mining and Semantic Search with Ontologies

Biomedical ontologies are not only used for data annotation but also serve as the backbone for semantic search and text mining applications. LLMs have been applied to enhance these tasks by providing sophisticated language understanding that can map unstructured text to structured ontology terms. For example, ref. [13] developed an LLM-assisted system to mine enzyme-substrate interactions from thousands of research articles. The system successfully mapped extracted relationships to standardized ontology identifiers, achieving high precision and recall.

In another study, Groza et al. evaluated GPT models for phenotype concept recognition, demonstrating that with properly engineered prompts, LLMs can identify and normalize ontology terms in clinical narratives [59]. This capability is crucial for applications such as electronic health record (EHR) annotation, where consistency and accuracy in labeling clinical concepts can directly affect downstream analyses. The LLM’s capacity to capture context—such as understanding that “heart attack” and “myocardial infarction” refer to the same phenomenon—significantly enhances the quality of text mining outputs compared to traditional rule-based systems.

A particular strength of LLMs in semantic search and text mining is their ability to capture synonymy and paraphrase, which is especially important in biomedicine where multiple terms may describe the same concept (e.g., “heart attack” vs. “myocardial infarction”). Studies have shown that with carefully designed prompts or fine-tuning, LLMs can normalize such variations to standardized ontology identifiers, thereby improving recall and consistency in text mining pipelines [59]. However, ambiguity remains a critical challenge: biomedical language also contains terms that are lexically similar but semantically distinct (e.g., “hypertension” vs. “hypotension,” or “angiogenesis” vs. “angioplasty”). While LLMs can often disambiguate these cases using surrounding context, evaluations indicate that accuracy is variable and dependent on prompt design, domain adaptation, and ontology grounding [25,26]. This underscores the importance of coupling LLM-driven extraction with ontology-based validation, so that ambiguous terms are resolved against curated knowledge sources rather than left to probabilistic inference alone.

Moreover, LLMs facilitate semantic search by interpreting natural language queries and translating them into ontology-guided search parameters. Systems like the Phenomics Assistant allow users to ask complex biomedical questions in plain language, while the LLM uses the underlying ontology to fetch accurate and contextually relevant answers [60]. This represents a shift from keyword-based search to concept-based retrieval, enabling more intuitive access to large biomedical databases. Such approaches enhance not only the speed but also the interpretability of the search process, making biomedical literature more accessible to researchers.

Another major area where LLMs intersect with bio-ontology research is in text mining—extracting knowledge from unstructured text to either enrich ontologies or to answer queries using ontological knowledge. The biomedical literature and clinical notes contain a wealth of information that could augment ontology content (new terms, relationships, usage examples) if mined effectively. Likewise, users often want to query knowledge bases in natural language, requiring translation from text queries to ontology-based answers. LLMs, with their powerful natural language understanding and generation capabilities, are increasingly being applied to these tasks of knowledge extraction and semantic search.

One line of research uses LLMs to generate novel associations or hypotheses from text and then leverages ontologies to validate or contextualize them. Hamed and Lee (2025) present an approach where ChatGPT was prompted to generate disease-centric associations—linking diseases to related drugs, symptoms, and genes—by drawing on its embedded knowledge [61]. For example, given a disease name, the LLM might list possible symptom manifestations or therapeutic drugs. These generated associations were then systematically verified against biomedical ontologies and databases. The results were intriguing: for identifying known terms, ChatGPT achieved high accuracy (e.g., 90%+ for disease names and drug names, around 88–98% for gene names), indicating that the LLM has memorized a lot of biomedical entities. However, it struggled with less distinct categories like symptoms (only  50–60% accuracy), possibly due to the ambiguity and variability in symptom descriptions. When it came to linking diseases with other entities, about 89–91% of the disease-drug and disease-gene associations ChatGPT proposed were confirmed by literature sources or existing ontologies.

This is a remarkably high coverage, suggesting LLMs can efficiently surface plausible relationships that largely overlap with known biomedical knowledge. On the other hand, some associations were incorrect or had no support in the literature, highlighting the hallucination problem—the model sometimes outputs factually incorrect links. A particular limitation noted was that ChatGPT often produced ontology IDs for terms that were incorrect or did not exist (From Knowledge Generation to Knowledge Verification: Examining the BioMedical Generative Capabilities of ChatGPT). For instance, it might give a DrugBank or DOID identifier that looked valid but was actually made-up. This emphasizes the need for a verification step: the authors’ pipeline caught these by cross-checking the terms and IDs against reference ontologies (Disease Ontology, ChEBI for chemicals, etc.). RAG-style normalization that retrieves canonical ontology entries before generation can further improve accuracy (e.g., REAL) [62].

Interestingly, the study found that the associations ChatGPT generated tended to be ones present in relatively recent literature (past 5 years), hinting that the model’s knowledge is skewed toward newer or more commonly discussed findings [61]. In summary, this work illustrates a potent combination: use LLMs to generate candidate biomedical knowledge triples, but anchor them to ontologies for validation. The ontology acts as a safeguard and a source of truth to filter out spurious model outputs. This kind of ontology-grounded text mining can accelerate hypothesis generation while maintaining reliability by continuously checking the LLM’s output against curated knowledge sources.

LLMs have also been applied directly to classic biomedical text mining tasks such as named entity recognition (NER) and concept normalization. An example is recognizing phenotype terms in text and mapping them to an ontology like the Human Phenotype Ontology (HPO). Traditional approaches use sequence taggers or dictionary matching for this, but Groza et al. evaluated GPT-3.5 and GPT-4 on the phenotype concept recognition task [59]. They experimented with prompting the LLMs in various ways (with different instructions and examples) to see if the models could identify phenotype mentions in both biomedical research abstracts and clinical notes. The best results came from using in-context learning, where the prompt included examples of text with annotated phenotypic terms, effectively guiding the model on how to respond. With this technique, GPT-4 achieved a document-level F1 score of 0.58 on recognizing HPO terms in scientific abstracts, and 0.75 F1 on clinical notes. At the mention level (individual term detection), performance reached about 0.70 F1, which actually surpassed the current best traditional tool for this task [59]. This is notable—it suggests that a suitably prompted LLM can rival or exceed specialized NER systems, likely because it has seen many ways phenotypic concepts are described in its training data. However, there were important caveats. Without providing example annotations in the prompt (i.e., zero-shot usage), the LLM’s performance was significantly worse than the conventional approaches.

This indicates that, out-of-the-box, even powerful LLMs might not reliably perform fine-grained extraction without some guidance. Moreover, the authors pointed out the non-deterministic nature of LLM outputs: the same prompt and text could yield slightly different answers on different runs [59]. This lack of consistency poses challenges for reproducibility in an extraction pipeline. The cost of using large models for many documents was also highlighted as a concern. Thus, while GPT-4 showed impressive capability in concept recognition, the study concludes that using LLMs for such tasks is “challenging” in practice due to variability and expense, unless the scope is constrained. Interestingly, they note that GPT-4’s performance was strongest when the task was restricted to a subset of the ontology that was expected (i.e., the model knew which specific terms might be relevant). This hints that if an ontology or context is provided, LLMs can be much more precise—aligning with the idea of ontology-guided prompting. In essence, LLMs can be very effective text miners for ontology terms if used with careful prompt design and with the ontology’s context, but one must handle their unpredictability and ensure results are verified, ideally against ground-truth ontologies or expert annotations.

In addition to phenotype recognition with GPT models [59], robust biomedical entity linking pipelines (e.g., BioSyn, SapBERT, BERN2) and annotation services (NCBO Annotator/BioPortal) remain essential for normalizing LLM outputs to stable identifiers [24,63,64,65]. A growing line of work targets automatic normalization of rare-disease phenotypes to HPO with fine-tuned LLMs and RAG pipelines, reporting substantial gains over zero-shot prompting [66,67]. Complementary analyses show that prior ontology knowledge and retriever design strongly influence success rates for phenotype linking, and propose simplified retrieval schemes that improve stability in practice [68,69]. These results collectively support pairing LLMs with ontology-aware retrieval for robust normalization workflows.

Beyond extraction, LLMs are enhancing how users search and interact with ontological knowledge. One emerging application is using conversational LLMs as natural language interfaces to biomedical knowledge graphs (which are often built on ontologies). For example, the Phenomics Assistant is a prototype chat-based system that allows users to query the Monarch Disease Phenotype Knowledge Graph in everyday language [60]. Behind the scenes, an LLM interprets the user’s question, retrieves relevant entities/edges from the underlying ontology-based knowledge graph, and formulates a helpful answer. Such systems leverage the semantic structure of ontologies (to find precise answers) while using the LLM to handle the nuances of human language in the query and response. This opens up ontologies to a broader audience—researchers or clinicians can ask complex questions (e.g., “Which genes are implicated in both disease X and phenotype Y?”) without knowing formal query languages, and the LLM can translate that into an ontology query and back to English results.

Early reports indicate that users find this significantly more intuitive than formulating queries via SPARQL or keyword search, though ensuring the accuracy of the answers is paramount (the LLM must not hallucinate relations that aren’t in the knowledge graph). Another related use of LLMs is in semantic search over literature or databases: instead of keyword matching, LLMs (or their embeddings) can capture the meaning of a query and find relevant information even if exact terms differ. For instance, an LLM could be prompted with a research question and asked to return relevant ontology concepts or articles. Or the embeddings of an LLM could be used to cluster and retrieve documents by concepts. Integrating ontologies in this process (by mapping text to ontology terms) enhances the relevance of results—essentially, the ontology provides a backbone of meaning for the LLM to latch onto.

A practical challenge that has emerged in LLM-assisted text mining and semantic search is reproducibility. Even when using identical prompts and inputs, stochastic decoding parameters can cause subtle variations in outputs—such as differences in extracted entity boundaries or chosen ontology identifiers. This variability complicates the reproducibility required for ontology-driven pipelines, where deterministic behavior is essential for auditability and version control. Several mitigation strategies have been proposed to address this issue, including ontology-guided prompting that anchors model outputs to canonical terms, deterministic or low-temperature sampling to reduce randomness, and fixed random seeds to ensure consistent decoding. When combined with post-generation validation against reference ontologies, these strategies can substantially improve stability while retaining the semantic flexibility that makes LLMs effective for biomedical text understanding.

### 3.4. Knowledge Graphs and Ontology Integration

Knowledge graphs (KGs) are another powerful tool for representing biomedical data. Typically, KGs incorporate ontologies as their schema, enabling structured, semantic connections between entities such as genes, diseases, and drugs. Recent studies have explored the integration of LLMs into KG construction and curation to improve both the quality and coverage of biomedical knowledge bases.

Soman et al. introduced a KG-optimized prompting framework in which an LLM is conditioned on subgraphs extracted from a biomedical KG [70]. This approach enhances the factual accuracy and context of the LLM-generated responses, ensuring that answers are backed by verifiable information. Similarly, Callahan et al. detailed an open-source KG ecosystem that benefits from LLM-assisted curation. In this system, LLMs help to populate the KG by extracting relationships and entity mentions from unstructured text and aligning them with the ontology-driven structure of the KG [71]. These integrated approaches reduce the incidence of “hallucinated” outputs by grounding the LLM’s responses in the curated, structured knowledge of the KG.

In addition, the use of LLMs has been extended to interactive KG construction, where curators can engage in a dialogue with the model to refine or extend the KG. For example, in an experimental setup, curators provided natural language feedback on the KG’s content, and the LLM adjusted its suggestions accordingly. Such interactive systems not only speed up the curation process but also improve the interpretability of the KG by linking each addition to a clear rationale based on literature or ontology guidelines. The success of these methods points to a future where KGs and ontologies are continuously updated and maintained with minimal human intervention, driven by real-time AI assistance.

Traditionally, KGs are built and maintained through symbolic reasoning and manual curation, ensuring logical consistency and enabling powerful inference over structured data. However, this symbolic approach can be limited in scalability and in its ability to capture nuanced associations expressed in unstructured biomedical text.

LLMs complement symbolic reasoning by bridging this gap between unstructured and structured data. On one hand, they can extract candidate entities and relations directly from biomedical literature or clinical text and propose them as additions to existing KGs [72]. On the other hand, when paired with ontologies, LLMs can assist in generating contextual definitions or mapping ambiguous terms to canonical KG nodes, thereby improving interoperability across heterogeneous data sources. Importantly, neuro-symbolic approaches that combine LLMs with formal reasoning systems are emerging as a promising hybrid paradigm: the LLM generates candidate triples or definitions, while ontology-based reasoning engines validate these outputs against logical constraints, filtering out inconsistent or spurious information [53].

To illustrate how symbolic reasoning complements neural generation, consider a case where an LLM proposes the RDF triple (BRCA1, associatedWith, Breast Cancer) during knowledge graph construction. Before acceptance, a reasoning engine grounded in the Disease Ontology and Gene Ontology can validate this assertion by checking whether the predicate associatedWith is permissible between entities of type gene and disease, and by confirming that both identifiers resolve to canonical entries. If the model instead produces an invalid relation—such as (BRCA1, causes, Cell Cycle Process)—the reasoner flags a domain or range violation, prompting curator review or automatic rejection. This workflow demonstrates how neuro-symbolic integration enforces factual integrity: the LLM supplies candidate knowledge, while ontology-based reasoning ensures that only logically coherent and biologically valid triples are incorporated into the graph.

This integration matters because biomedical AI often depends on reasoning across large, heterogeneous datasets. For example, drug repurposing studies require linking molecular interaction data to phenotypic outcomes, while clinical decision support may involve combining EHR data with curated knowledge bases. By pairing the generative and semantic capabilities of LLMs with the rigor of symbolic reasoning, researchers can build knowledge graphs that are more comprehensive, adaptable, and capable of supporting complex queries that go beyond keyword search.

Biomedical knowledge graphs and schemas (Hetionet, RTX-KG2, the Biolink Model, and the Monarch KG) provide the semantic backbone needed to constrain LLM outputs and enable interoperability [20,73,74,75]. Retrieval-augmented generation conditioned on KG subgraphs has shown improved factual grounding [70].

Recent work published in 2025 further highlights the rapidly evolving role of LLMs in bio-ontology research and related domains. Hier et al. [52] analyzed why LLMs fail to map ontology terms to identifiers in resources such as HPO and GO, identifying features like identifier familiarity and ontology “deserts” as strong predictors of failure. In the area of relation extraction, the RELATE framework [53] integrates LLM predictions with ontology constraints and reranking strategies to ensure that extracted biomedical relations conform to valid ontological predicates. Ontology alignment has also advanced through methods such as MILA [35], which leverage hierarchical prompts and retrieval strategies to improve mapping precision across ontologies. For ontology enrichment, Kollapally et al. [76] demonstrated a pipeline that uses LLMs to propose new ontology terms and definitions from biomedical corpora, illustrating a practical workflow for expanding existing resources. At the level of knowledge graph integration, Mavridis et al. [72] showed how LLMs can be combined with vector-based retrieval to generate RDF triples aligned with SNOMED CT, supporting semantic web interoperability. Finally, systematic benchmarking efforts [77,78] provide critical evaluations of LLM performance on biomedical tasks, underscoring both the promise and variability of current approaches. Together, these recent studies reinforce the importance of continued innovation and highlight emerging strategies that bring LLM-driven ontology engineering closer to practical deployment.

Several studies report end-to-end pipelines where LLMs extract entities/relations from clinical or biomedical text and instantiate them in ontology-backed KGs, including sepsis-focused graphs and biosample metadata annotation [79,80,81]. A recent survey catalogs design choices for LLM-based KG construction across retrieval, prompting, and validation layers [29]. Domain-specific KGs (e.g., RNA-KG) further demonstrate how explicit ontological schemas constrain extraction and integration [82]. Change-management languages for ontologies and KGs (e.g., KGCL) are beginning to formalize curator-facing edits when LLMs propose additions, improving traceability [83].

**Figure 1:**
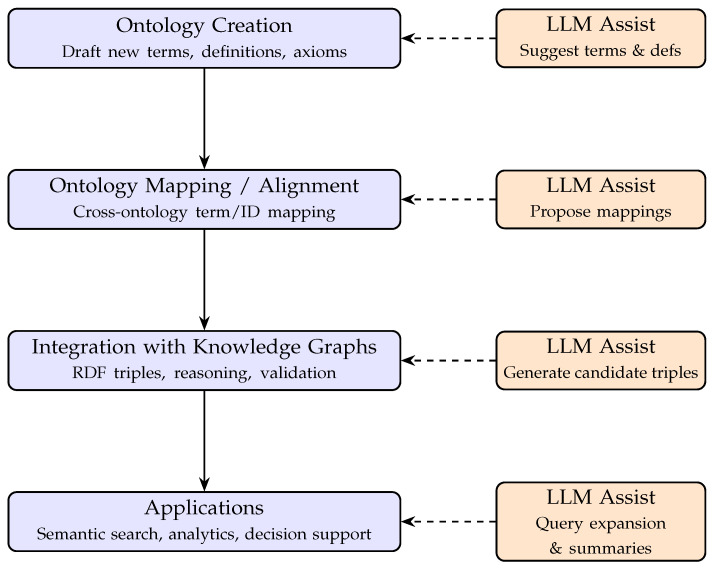
Vertical schematic of the ontology pipeline (Creation → Mapping → Integration → Applications) with LLMs assisting at each step. Dashed arrows indicate supportive roles, while solid arrows represent the traditional curator-driven workflow.

Taken together, these applications illustrate how LLMs can assist at multiple points in the ontology lifecycle, from the creation of new terms and definitions to mapping across ontologies, integration into knowledge graphs, and support for downstream applications such as semantic search and clinical decision support. To provide a concise overview of this workflow, Figure 1 presents a schematic illustration of the ontology pipeline, highlighting where LLMs contribute as accelerators in an otherwise curator-driven process.

To help synthesize the wide range of applications discussed above, we provide a summary table (Table 1) that maps key ontology-related tasks to representative LLM-based approaches, highlighting their main strengths and weaknesses. This overview is intended to give readers a concise reference point and a “big picture” view of how LLMs are currently being integrated into bio-ontology research. By organizing the field in this way, the table underscores both the diversity of tasks where LLMs are already proving useful—such as ontology creation, mapping, semantic search, and knowledge graph integration—and the persistent limitations that necessitate human oversight and further methodological innovation.

### 3.5. Comparative Analysis: Scalability, Maturity, and Limitations

While recent studies have demonstrated a range of innovative approaches for ontology creation, mapping, and integration, their levels of maturity and practical scalability vary considerably. Classical ontology matchers such as AgreementMakerLight (AML) and LogMap remain the most reliable for production-scale use, particularly in environments requiring deterministic behavior, transparent reasoning, and auditability. Their reliance on lexical and structural similarity ensures reproducibility but often limits their capacity to capture nuanced semantic relationships, especially when ontologies differ in language, granularity, or scope.

Hybrid methods that combine deterministic heuristics with LLM-based semantic refinement—such as MapperGPT, MILA, and SPIREX—represent an emerging middle ground. These systems have achieved measurable gains in recall and semantic coverage while maintaining manageable precision levels through selective prompting and human-in-the-loop validation. Among these, MILA stands out as one of the first frameworks to demonstrate near-production scalability, achieving competitive F1 scores across multiple biomedical alignment tasks while reducing computational overhead by limiting LLM calls to ambiguous cases. However, even these hybrid systems depend on curator oversight and lack standardized evaluation of throughput, latency, and total cost of ownership.

By contrast, purely LLM-driven pipelines—those relying solely on generative or retrieval-augmented reasoning for ontology alignment—remain largely proof-of-concept. They exhibit strong linguistic and contextual understanding but tend to generate spurious mappings, inconsistent identifiers, and variable outputs across runs. These characteristics constrain their suitability for regulated biomedical knowledge systems that require strict version control and traceability.

A comparison of scalability, maturity, and limitations across major methodological categories is summarized in Table 2. This synthesis highlights that hybrid systems currently offer the best trade-off between automation, semantic depth, and governance readiness, while classical methods remain indispensable for ensuring logical soundness and reproducibility.

The comparative insights summarized in Table 2 also illuminate several key research gaps. First, while rule-based systems remain stable and reproducible, they lack semantic depth and contextual adaptability, underscoring the need for hybrid architectures that combine symbolic reasoning with neural contextualization. Second, the partial scalability of hybrid LLM-assisted frameworks reveals the absence of standardized evaluation benchmarks that capture both accuracy and curator effort across diverse ontology domains. Third, the limited deployment of fully LLM-based pipelines highlights unresolved issues surrounding reproducibility, bias mitigation, and ontology-constrained grounding. Collectively, these observations indicate that the next phase of research should focus on developing auditable, ontology-aware evaluation frameworks and cross-domain benchmark suites to assess real-world scalability, interpretability, and bias resilience in LLM-augmented ontology engineering.

In summary, while LLMs offer substantial gains in semantic flexibility and contextual understanding, their current implementations are best positioned as augmentative components within hybrid frameworks rather than replacements for symbolic or rule-based systems. Sustained progress toward scalable, auditable ontology integration will depend on tighter coupling between LLM reasoning, ontology-grounded validation, and transparent benchmarking.

### 3.6. Milestones in LLM-Assisted Ontology Engineering: Strengths and Trade-Offs

Over the past two years, several systems have marked important milestones in the application of large language models (LLMs) to ontology engineering. Each milestone demonstrates both a conceptual advance and practical constraints, underscoring the field’s rapid evolution from exploratory prototypes to partially deployable frameworks.

SPIRES and DRAGON-AI (2023–2024) introduced structured prompting and iterative refinement for ontology term generation and curation, showing that LLMs can assist with definition writing and semantic normalization [48]. Pros: demonstrated early success in aligning generated content with ontology design patterns. Cons: required heavy human supervision, lacked reproducibility testing, and did not integrate logical validation during generation.

MapperGPT (2023) was among the first to formalize LLM-assisted ontology alignment by combining classical lexical matchers with contextual reasoning [46]. Pros: improved recall for semantically distant term pairs and introduced the “semantic curator” paradigm. Cons: remained limited to small-scale test sets and did not quantify scalability, prompting variability, or curator time saved.

MILA (2024–2025) achieved a significant leap in scalability through a prioritized depth-first search strategy that invokes LLMs only for uncertain mappings [35]. Pros: demonstrated state-of-the-art F1 scores (0.83–0.95) across biomedical alignment tasks and reduced LLM query volume by over 90%. Cons: relied on heuristic pre-filtering and lacked longitudinal evaluation of maintenance costs and curator agreement.

RELATE and OntoTune (2025) represent emerging neuro-symbolic frameworks that couple LLM generation with ontology reasoning or self-training on hierarchical structures [53,57]. Pros: explicitly enforce logical consistency and begin to bridge symbolic and neural representations. Cons: still in early proof-of-concept stages, with limited benchmark reporting and unclear generalizability beyond the biomedical domain.

GenOM (2025) extends the use of LLMs to enrich sparse ontology labels with generated definitions prior to matching, highlighting progress in domain adaptation [58]. Pros: addresses data sparsity and improves recall. Cons: computationally intensive and vulnerable to factual drift in generative text.

Overall, these milestones illustrate a clear trajectory: from manually supervised generative tools to hybrid systems capable of measurable gains in scalability and precision. While early methods established feasibility, recent frameworks such as MILA and OntoTune demonstrate the potential for structured, ontology-grounded integration. Future research should continue to merge the interpretability of symbolic approaches with the contextual depth of neural reasoning to achieve scalable, auditable, and domain-robust ontology automation.

## 4. Domain-Specific BioLLMs: Challenges, Limitations, and Future Directions

### 4.1. Challenges

The integration of LLMs in bio-ontology research is still in its early stages, yet several promising avenues for future exploration have emerged.

#### 4.1.1. Data Scarcity and Quality

Biomedical language models often face a narrower and more specialized data pool compared to general-domain LLMs. While millions of biomedical articles exist, this is modest relative to the open-domain web text, and the content has domain-specific terminology and distributions. As a result, directly fine-tuning general LLMs (e.g., BERT or ELMo) on biomedical tasks yields poor performance, since such models were trained on general corpora (Wikipedia, books) and miss domain-specific vocabulary and style [84]. When evaluating BioLLMs for ontology-centric tasks, use domain benchmarks such as BLUE and BLURB to contextualize gains beyond general NLP metrics [16,85]. High-quality annotated data in biomedicine are especially scarce due to the cost and expertise required for labeling (e.g., clinical notes or biomedical literature) [86]. This scarcity of comprehensive, unbiased corpora and gold-standard annotations limits BioLLM performance and their ability to generalize. Researchers have alleviated this by curating domain-specific corpora (e.g., PubMed abstracts, clinical reports) for pretraining, but coverage gaps and uneven data quality (e.g., class imbalance, outdated information) remain persistent issues [86].

#### 4.1.2. Bias and Ethical Concerns

Despite their significant potential, LLMs come with several challenges that must be addressed to ensure their safe and effective use in bio-ontology research. Like all AI trained on human text, BioLLMs can inherit and even amplify biases present in biomedical literature or health records. This includes demographic biases (e.g., underrepresentation of certain genders or ethnic groups in clinical trials) that may lead to skewed model predictions [27]. LLMs are trained on large, heterogeneous corpora that may contain inherent biases, which can be inadvertently reflected in the ontology suggestions. For example, if certain diseases or demographics are underrepresented in the training data, the LLM may fail to propose relevant ontology terms for those areas. For instance, a recent evaluation found that GPT-4’s responses on clinical scenarios can perpetuate racial and gender biases present in the training data [27]. Such biases pose serious ethical concerns in medical applications, potentially affecting diagnostic or treatment suggestions. Researchers stress the need for rigorous bias evaluation and mitigation techniques in deploying LLMs for biomedical applications [83].

Privacy and data security are also of paramount importance, particularly when sensitive clinical data is involved. When using cloud-based LLM services, there is a risk that confidential information could be inadvertently exposed. Therefore, secure, on-premise solutions or robust de-identification protocols must be implemented. Lastly, the reproducibility and reliability of LLM outputs pose practical challenges. Variability in responses, due to the probabilistic nature of these models, can hinder consistent ontology curation. Many studies advocate for a human-in-the-loop approach, where expert curators validate and refine the LLM-generated content. Such practices are essential to ensure that AI-augmented ontologies maintain the high quality and precision required for biomedical research.

Despite their significant potential, LLMs come with challenges that must be addressed to ensure their safe and effective use in bio-ontology research. Like all AI trained on human text, BioLLMs can inherit and even amplify biases present in biomedical literature or health records. This includes demographic biases such as the underrepresentation of certain genders or ethnic groups in clinical trials, which can result in skewed model predictions or omissions in ontology coverage.

For example, cardiovascular disease has historically been studied predominantly in men, leading to symptom descriptions that emphasize male presentations (e.g., chest pain), while atypical symptoms more common in women (e.g., fatigue or nausea) are underreported [87]. An LLM trained on such biased data may therefore prioritize male-centric disease descriptors when generating ontology terms or mappings. Similarly, studies of dermatological conditions have shown that images and descriptions of skin disease are disproportionately based on lighter skin tones, causing diagnostic tools—and by extension ontology-driven annotation systems—to underperform on patients with darker skin [88].

Racial and ethnic disparities have also been documented in clinical datasets: for instance, pulse oximeters systematically overestimate oxygen saturation in Black patients, an error that propagates into EHR records and clinical decision-making [89]. If an LLM were to ingest and normalize such biased data into ontology terms, it could inadvertently reinforce inequities in downstream applications, such as phenotype recognition or clinical decision-support systems.

These examples highlight that bias in biomedical data is not merely theoretical but has real consequences that could be magnified by ontology engineering pipelines. Rigorous bias evaluation, mitigation techniques (e.g., balanced training corpora, fairness audits), and continuous human oversight are therefore critical to ensure that BioLLMs support equitable and accurate biomedical knowledge representation [27].

#### 4.1.3. Hallucination and Factual Consistency

In the biomedical domain, factual precision is paramount—an incorrect drug dosage or a hallucinated comorbidity in a generated report can have life-threatening consequences. However, large language models are prone to hallucinations: they may produce confident results that are completely fabricated or not supported by any source [25]. Ensuring factual consistency is a key challenge, as BioLLMs must align with established medical knowledge and the latest evidence. Models like ChatGPT have been observed to sometimes generate citations from the non-existent literature or incorrect medical information, which is particularly dangerous in clinical decision support. Techniques for reducing hallucinations (e.g., grounding the model’s responses in verified databases or forcing it to cite sources) are actively being investigated, but achieving consistent truth remains difficult. As demonstrated by Reese et al., even state-of-the-art models can sometimes propose plausible but incorrect ontology entries or mappings. Such errors, if unchecked, could propagate into biomedical knowledge bases with potentially serious consequences [26].

In ontology construction, an LLM might confidently generate a new disease class such as “Chronic Lyme Spectrum Disorder” or assign a plausible but non-existent identifier (e.g., a fabricated DOID or SNOMED code). If incorporated without expert review, such terms could propagate into downstream annotation pipelines, leading to false mappings in patient records or erroneous enrichment analyses in research studies. Similarly, an LLM might assert a relationship between two existing entities that does not exist in the literature—for instance, linking a gene to a disease based on a spurious co-occurrence. In clinical contexts, these hallucinations could mislead decision-support systems by suggesting non-validated associations, potentially influencing diagnosis or treatment recommendations. These risks underscore why human-in-the-loop validation and ontology-grounded verification steps are indispensable when incorporating LLM outputs into biomedical knowledge frameworks [25,26].

The MILA system recently reported F1 scores of up to 0.948 on NCIT–DOID ontology alignment tasks, outperforming leading unsupervised systems, and achieved a reduction in LLM query counts of >92% compared to a naïve RAG baseline. While this demonstrates strong semantic accuracy and cost efficiency for a hybrid workflow, it still relies on heuristics to limit prompts, and the evaluation does not fully detail long-term curator time savings or real-world deployment constraints. By contrast, MapperGPT reports improved accuracy over lexical baselines in challenging ontology-mapping scenarios, but lacks published large-scale deployment metrics or comprehensive error analyses, indicating that many LLM-based methods currently remain at the proof-of-concept stage. These findings highlight that, although LLM-augmented ontology tools are advancing rapidly, practitioners must still guard against issues of reproducibility, hallucination, and lack of longitudinal performance data.

#### 4.1.4. Computational Challenges in Fine-Tuning

The biomedical NLP community must often work with models that are billions of parameters in size, which makes training and fine-tuning resource-intensive. Adapting a general large model to the biomedical domain (or training a new BioLLM from scratch) can require enormous computing power and memory. For example, recent studies report using dozens of high-end GPUs for extended periods to continuously pre-train a 13 billion-parameter biomedical model [77]. The expense and carbon footprint of such large-scale training runs are non-trivial, creating a barrier for academic and clinical researchers without access to industrial-level compute. Additionally, biomedical text can be lengthy (research articles, clinical notes) and contain long-range dependencies, pushing the limits of LLMs’ context windows and memory. Efficient fine-tuning is also challenging: naively updating all weights of a giant model on domain data is not only slow and costly but risks overfitting or catastrophic forgetting of general language abilities. There is ongoing work on lightweight fine-tuning methods (e.g., adapters or LoRA) to reduce computational burden, but scaling these to very large models while maintaining performance is an open problem. In summary, the size and computational demands of state-of-the-art BioLLMs hinder rapid experimentation and deployment, especially for smaller organizations.

## 5. Limitations

Despite their potential, the application of LLMs to ontology engineering faces important limitations. These can be grouped into three broad categories: data issues, algorithmic issues, and clinical application issues.

### 5.1. Data Issues

LLMs inherit the biases and noise present in their training corpora, which often underrepresent certain populations, rare diseases, or non-English biomedical literature [90]. In the context of ontologies, this can result in incomplete coverage or skewed term proposals that reflect dominant trends in the literature rather than the full diversity of biomedical knowledge. In addition, many corpora used for training are not curated for biomedical accuracy, meaning that spurious or outdated information may surface as candidate ontology content [26]. Another challenge lies in grounding: LLMs can generate plausible-looking definitions or synonyms that are not linked to stable identifiers, creating integration problems for downstream use.

### 5.2. Algorithmic Issues

At the algorithmic level, hallucination remains a major limitation. LLMs may fabricate ontology terms, definitions, or relationships that appear credible but lack any biomedical grounding, potentially polluting ontologies if not rigorously checked. While prompting strategies and fine-tuning can reduce these risks, reproducibility remains a challenge: identical prompts may yield different outputs across runs or model versions [77]. Moreover, the cost and computational demands of inference at scale create barriers to routine adoption in ontology curation workflows [91]. Evaluation is another unresolved issue: existing NLP metrics (e.g., BLEU, F1) do not adequately capture ontology-specific outcomes such as logical consistency, interoperability, or curator time saved.

### 5.3. Clinical Application Issues

Finally, the translation of LLM-assisted ontology methods into clinical and translational research raises unique concerns. Bias in biomedical data can amplify disparities in downstream applications, such as phenotype annotation or clinical decision support [92]. Even subtle errors in mapping or definition generation may propagate through knowledge graphs and affect clinical inference. In addition, integrating LLM workflows into established ontology governance structures—such as those promoted by the OBO Foundry [8] requires careful design of validation pipelines and curator training. Without strong oversight, LLM-assisted curation risks undermining the trustworthiness and interoperability that ontologies are meant to ensure.

Overall, while LLMs offer promising accelerations in ontology creation and mapping, these limitations underscore the need for hybrid human–machine pipelines, rigorous evaluation frameworks, and strong governance to ensure reliability, fairness, and clinical safety.

To translate these limitations into actionable guidance, several best practices are recommended for practitioners integrating LLMs into ontology workflows. First, mandatory identifier validation against reference ontologies (e.g., GO, HPO, MONDO) should be enforced to prevent fabricated or misaligned entities from entering production systems. Second, prompt templates, model parameters, and retrieval sources should be logged for each generation event to ensure full auditability and reproducibility across ontology releases. Third, automated reasoning checks should be incorporated into the validation pipeline to detect logical inconsistencies or domain/range violations early. Finally, implementing version-controlled review workflows—where each LLM suggestion is traceable to a prompt, timestamp, and curator decision—can provide transparency and maintain community trust. Embedding these practices within ontology governance processes bridges the gap between experimental AI research and sustainable, auditable biomedical knowledge management.

In summary, addressing these challenges requires a coordinated shift toward transparency, reproducibility, and ontology-grounded validation. Future research should prioritize the standardization of evaluation benchmarks to ensure comparability across LLM-assisted ontology tools, the development of ontology-grounded retrieval-augmented generation (RAG) pipelines that reduce hallucination and improve factual grounding, and the implementation of systematic bias audits to monitor representational fairness across biomedical domains. Strengthening these foundations will be essential for advancing LLMs from promising prototypes to trustworthy, production-ready components in ontology engineering workflows.

### 5.4. Future Directions

#### 5.4.1. Domain Adaptation and Continuous Learning

Given the aforementioned data limitations, one crucial direction is improving the domain adaptation of BioLLMs. Domain-adaptive pretraining—starting from a general model and further training it on biomedical corpora—has already shown significant gains, and future models will benefit from even more targeted pretraining strategies [84]. In fact, fully domain-specific models like PubMedBERT (trained from scratch on PubMed texts) can outperform models that were only fine-tuned on biomedical data, demonstrating the value of in-domain representations. Building on this, continuous learning techniques aim to keep BioLLMs up-to-date with the latest medical knowledge. The biomedical field evolves quickly (e.g., new diseases, emerging therapies), so a model frozen to a 2023 knowledge cutoff will rapidly become outdated. Future BioLLMs are likely to adopt continual training regimes, periodically ingesting new publications and guidelines without forgetting prior knowledge. This could involve scheduled refresh training, or architectures that can expand to accommodate new facts. Another aspect is lifelong learning on the user side: BioLLMs might personalize and refine themselves as they interact with clinicians or researchers (while preserving privacy). Overall, the goal is a BioLLM that remains a living knowledge base, constantly learning from new data. Research in domain continual learning supports this: for example, techniques have been proposed to progressively fuse new biomedical knowledge sources into a model without retraining from scratch [93]. Such approaches will help BioLLMs maintain state-of-the-art performance as the domain grows.

#### 5.4.2. Integration with Ontologies and Knowledge Bases

A promising way to enhance biomedical LLMs is to integrate them with structured domain knowledge, such as biomedical ontologies, knowledge graphs, and database-backed clinical facts. Unlike a purely statistical model, an ontology-backed LLM can cross-check its outputs against a curated knowledge base, improving accuracy and interpretability. One approach is retrieval-augmented generation: before answering a query, the BioLLM can retrieve relevant facts (e.g., a drug’s properties from DrugBank, or relations from UMLS) and use them as context. This method has been shown to significantly ground model responses in truth-for example, injecting a large biomedical knowledge graph into the prompt can produce answers that remain aligned with established medical knowledge [70]. Another approach is to have the LLM’s outputs post-processed or validated by symbolic rules (for example, ensuring a generated treatment plan doesn’t violate clinical guidelines encoded in an expert system). There is evidence that hybrid systems can outperform LLMs alone: a neuro-symbolic model that links generated text to an ontology (UMLS) yielded improved accuracy in recognizing and normalizing cancer-related entities from clinical notes [94]. Beyond using knowledge to aid LLMs, the converse is also an exciting direction: using LLMs to assist with ontology curation. Future BioLLMs might act as intelligent assistants for maintaining and extending biomedical ontologies, suggesting new relationships or definitions based on emerging literature. Tight integration between BioLLMs and structured knowledge—potentially via tools for ontology editing and querying—could thus both make LLM outputs more reliable and help keep knowledge bases up-to-date.

#### 5.4.3. Evaluation Metrics and Benchmarks for BioLLMs

As BioLLMs become more sophisticated, it is crucial to evaluate them on criteria that matter for biomedical tasks. General NLP benchmarks (GLUE, etc.) are not sufficient, so the community has developed domain-specific benchmarks. One example is BLURB (Biomedical Language Understanding and Reasoning Benchmark), a suite of biomedical NLP tasks (such as named entity recognition, relation extraction, question answering, etc.) built to assess BioLLMs across a broad range of capabilities [16]. These benchmarks provide standardized datasets and performance metrics (accuracy, F1, etc.) that reflect the challenges unique to biomedical text. In addition, challenge datasets like BioASQ (for biomedical question answering) and the MEDIQA series focus on clinical QA and entailment, pushing models to demonstrate factual correctness and reasoning in medical contexts. Going forward, we anticipate new evaluation metrics tailored to BioLLMs: for example, measuring clinical relevance of an answer, or factuality scores that penalize any medical hallucination. There is also a need for human evaluation by domain experts—e.g., physicians rating the usefulness and safety of a model’s suggestions. Benchmarking BioLLMs on multi-step reasoning (can the model interpret lab results and then suggest a diagnosis?) and on ethical criteria (does the model’s output align with medical ethics and patient privacy requirements) will likely become standard. Creating leaderboards and shared tasks around these evaluations will drive progress. In summary, specialized benchmarks and metrics are evolving in parallel with BioLLMs to ensure we can rigorously track improvements and shortcomings in real biomedical applications [16,84].

Beyond conventional NLP measures such as F1 or BLEU, ontology-centered evaluation demands metrics that capture real curation value and logical soundness. Domain-specific criteria could include (i) curator time saved—quantifying reductions in manual editing or review effort; (ii) validated suggestion rate—the proportion of LLM-generated terms, definitions, or mappings accepted after expert review; (iii) identifier fidelity rate—the percentage of outputs correctly grounded to canonical ontology IDs; and (iv) logical consistency rate—the share of new axioms that pass reasoning checks without contradiction. Additional indicators, such as reproducibility across runs and audit trace completeness, can further assess the reliability of AI-assisted pipelines. Collectively, these metrics move evaluation beyond linguistic accuracy toward operational performance, offering a clearer picture of how effectively LLMs enhance ontology engineering in practice.

#### 5.4.4. Hybrid Neural–Symbolic Reasoning Models

Finally, an important future direction is the development of hybrid models that combine the statistical power of large neural LMs with the robustness of symbolic reasoning. Pure neural models excel at language understanding and generating fluent text, but they lack explicit reasoning chains and can struggle with logical consistency. In the biomedical domain, there is enormous value in integrating LLMs with systems that perform symbolic inference (for example, a logic reasoner verifying that a treatment plan conforms to all known contraindication rules). Hybrid approaches could involve an LLM working in tandem with a knowledge graph reasoner: the LLM proposes a hypothesis or answer, and a symbolic module validates it against a knowledge base or deduces consequences. Early work in this direction is promising—for instance, combining an LLM with ontology-based reasoning was shown to improve the recognition of entities and their relationships in clinical text, effectively boosting the model’s understanding by enforcing consistency with known medical ontologies [94]. Another potential is to use LLMs to translate natural language queries into formal queries (for databases or reasoners) and then convert the results back into readable answers, marrying conversational ability with precise logical querying. By leveraging symbolic AI (which by nature is transparent and rule-based) alongside neural LMs (which are flexible and data-driven), such neuro-symbolic systems could achieve the best of both worlds. They would be better at handling complex decision-making tasks in medicine that require step-by-step reasoning or adherence to formal guidelines. In the long term, we expect biomedical AI to incorporate more of these hybrid architectures, ensuring that the powerful language generation of BioLLMs is always channeled through a safety net of factual and logical correctness.

#### 5.4.5. Interactive and Collaborative Curation Tools

Emerging platforms are poised to feature interactive interfaces that enable ontology curators to engage in real-time dialogue with large language models (LLMs). Unlike static, one-shot prompts, these tools will support iterative refinement, allowing curators to request AI-generated ontology components, review suggestions, and provide contextual corrections. This human–AI collaboration can lead to higher-quality ontologies while reducing the manual burden on curators.

Current research is already exploring conversational agents for ontology curation, and this trend is expected to grow. However, designing effective human–AI interactions for real-time ontology editing presents several challenges. A key concern is user trust, ontology engineers must have confidence that AI-generated suggestions will not compromise the integrity of their ontology. If the system is opaque or prone to errors, experts may hesitate to rely on its assistance. Interface complexity is another issue; ontology editing is inherently intricate, and introducing an AI assistant must be done intuitively to avoid overwhelming users. Striking the right balance between automation and user control is crucial—users should be able to override AI-generated suggestions while still benefiting from AI-assisted insights.

Early experiments with conversational ontology curation tools suggest that users require iterative refinement and guidance to achieve useful outcomes. For instance, an LLM may generate an initial set of class definitions or competency questions that are only partially correct, requiring multiple rounds of adjustments. Prototypes such as OntoChat are already demonstrating the potential of conversational agents to translate user stories into ontology requirements through interactive prompting [95]. Future tools will expand on this, incorporating adaptive learning loops where the AI refines its suggestions based on user accept/reject feedback. For example, if a domain expert corrects an AI-generated classification, the system could update its internal model or retain that feedback for future interactions.

Research in UI/UX for AI-assisted ontology editing will explore visual mechanisms to present AI recommendations such as highlighting confidence scores or alignment with existing ontology rules so that users can easily evaluate them. Additionally, collaborative platforms may allow multiple experts to interact with the AI in shared workspaces, with the system facilitating discussions and consolidating inputs. Ultimately, the future of ontology engineering is moving toward interactive, AI-augmented environments where AI assists in ensuring consistency and completeness, while human experts provide oversight, domain expertise, and final validation.

A further set of challenges relates to scalability, reproducibility, and adoption barriers that go beyond technical accuracy. First, the scalability of LLM-based workflows remains a practical obstacle: high computational demands for both training and inference limit their accessibility to well-resourced institutions, raising concerns about equitable adoption across the biomedical community [77,90]. The cost of inference at scale—particularly when processing millions of abstracts or clinical texts—can make routine ontology enrichment prohibitively expensive without careful optimization or model distillation [91]. Second, reproducibility remains a concern: identical prompts may yield different outputs across runs or model versions, complicating ontology development pipelines that depend on consistency and auditability [26]. Finally, integration into existing ontology curation workflows poses social and organizational challenges: ontology communities have long-established peer review, consensus building, and governance processes [8], and community-driven roadmaps emphasize sustained collaboration [96]. Introducing LLM outputs therefore requires new validation protocols, curator training, and governance frameworks. Without addressing these barriers—efficient inference, reproducible behavior, and integration into curator workflows—widespread adoption of LLM-assisted ontology engineering will remain constrained, regardless of technical advances.

Beyond efficiency, scalability, and bias, several important directions remain underrepresented in current discussions of LLMs for bio-ontology research. One critical area is explainability and interpretability: while LLMs can generate candidate ontology terms or mappings, their decision-making process is often opaque. Without transparent explanations, curators may struggle to evaluate why a given term was proposed or how a mapping was derived, limiting trust and adoption in high-stakes biomedical settings [97]. Developing interpretable LLM frameworks or coupling outputs with attribution methods will therefore be essential for ensuring curator confidence.

A second underexplored direction is the design of interactive and visual ontology editing environments that incorporate LLM assistance. Current tools such as OntoChat provide a conversational interface, but there is an opportunity for richer platforms that combine LLM-driven suggestions with visualization, collaborative editing, and real-time reasoning checks. Such tools could substantially reduce curator burden while improving transparency and engagement across ontology communities [98].

Finally, integration with the FAIR principles (Findable, Accessible, Interoperable, Reusable) remains a key frontier. For LLMs to support sustainable ontology ecosystems, their outputs must not only accelerate curation but also align with FAIR data practices, ensuring interoperability across heterogeneous datasets and reusability in downstream pipelines [99]. Addressing these dimensions will help ensure that LLM-assisted ontology engineering advances not only in speed and scale but also in transparency, usability, and long-term sustainability.

## 6. Conclusions

Large language models (LLMs) are beginning to transform bio-ontology research by automating routine yet labor-intensive tasks and providing new ways to integrate biomedical knowledge. Evidence from recent studies shows that LLMs can draft ontology terms and definitions, assist in ontology mapping, normalize biomedical text for semantic search, and enrich knowledge graphs with new relationships. These applications highlight their value as intelligent assistants that can accelerate knowledge integration across genomics, clinical phenotyping, and biomedical text mining. At the same time, limitations—including hallucination, bias, reproducibility concerns, and privacy risks—underscore that current models cannot replace expert curation. Instead, the most promising paradigm is one of augmented ontology engineering, where LLMs take on repetitive or large-scale information extraction tasks while domain experts retain oversight of validation and integration.

Looking ahead, several specific directions stand out as critical for the field:

- Hybrid neuro-symbolic approaches. One of the most promising avenues is combining the probabilistic power of LLMs with the logical rigor of ontologies and reasoning systems. Neuro-symbolic methods could allow LLMs to propose candidate terms and relations, while symbolic reasoners check consistency against established ontology axioms. Early work already shows that hybrid models outperform standalone LLMs in entity recognition and mapping tasks. Developing robust pipelines for ontology-grounded reasoning will help ensure factual reliability and logical coherence in AI-assisted ontology curation.
- Domain-specific evaluation and benchmarking. Generic NLP benchmarks are insufficient for biomedical ontology tasks, where the stakes include clinical safety and scientific validity. Future research must develop ontology-focused evaluation metrics that assess logical consistency, ontology coverage, and alignment with curated gold standards. Resources such as BLURB and BioASQ provide a foundation, but dedicated benchmarks for ontology creation, mapping accuracy, and definition quality will be needed to guide model improvement. Equally important is incorporating human expert evaluation, where domain specialists assess whether AI-generated ontology content is clinically meaningful and biologically accurate.
- Interactive and collaborative curation platforms. Ontology development is a community-driven process, and the next generation of tools should reflect this. Embedding LLMs into real-time, collaborative editing platforms could support curators by suggesting terms, drafting definitions, and surfacing candidate mappings while still allowing experts to accept, reject, or modify suggestions. Early prototypes such as conversational ontology editors demonstrate the potential of such systems. Expanding these into multi-user platforms—where groups of experts and AI collaborate simultaneously—could reduce workload, improve transparency, and accelerate consensus building.
- Bias detection and mitigation. Biomedical datasets carry known demographic biases, such as under-representation of women in cardiology studies or lighter skin tones in dermatology images. If uncorrected, these biases can propagate into ontology terms, definitions, and mappings, perpetuating inequities. Future work should focus on bias-aware LLM training and auditing, where generated ontology content is evaluated for representational fairness and coverage across diverse populations. This will require both technical solutions (balanced corpora, fairness audits) and sociotechnical frameworks that embed equity considerations into ontology development practices.
- Continuous learning and adaptability. Biomedical knowledge evolves rapidly, with new diseases, drugs, and molecular mechanisms emerging every year. Static models quickly become outdated. A key direction is developing LLMs capable of continual domain adaptation, ingesting new literature, clinical data, and curated ontologies without catastrophic forgetting. Coupled with incremental ontology updating workflows, such systems could ensure that biomedical ontologies remain current and responsive to emerging discoveries.
- Ethical and governance frameworks. Finally, as LLMs become more tightly integrated into biomedical knowledge infrastructures, questions of accountability, authorship, and governance will grow in importance. Policies are needed to define how AI-generated content is validated, attributed, and disseminated. Ontology communities may need to establish standards for documenting AI contributions, auditing decision-making, and ensuring that ethical safeguards are systematically applied.

While LLMs offer clear advantages in efficiency by accelerating ontology construction, mapping, and enrichment, it is important to emphasize that their outputs cannot be deployed in a fully unsupervised manner. Models may hallucinate terms, misassign identifiers, or reproduce biases present in training data, any of which can compromise the integrity of an ontology if left unchecked. For this reason, human oversight remains indispensable: domain experts are needed to validate proposed terms and relationships, ensure logical consistency, and safeguard clinical and biological accuracy. The most promising paradigm is therefore a human-in-the-loop workflow, where LLMs act as accelerators that reduce curator workload, while expert review guarantees reliability and trustworthiness of the resulting ontologies [25,26].

### 6.1. Actionable Recommendations for Practice (Next 12–18 Months)

- Adopt hybrid pipelines: pair LLM proposals with ontology reasoners and ID validators before acceptance; require curator sign-off for any new class, definition, or mapping.
- Track curator ROI: instrument workflows to log “minutes-per-accepted-edit” and reject reasons to quantify true time savings.
- Harden grounding: enforce identifier normalization (e.g., HPO/GO/DO/MONDO) and fail closed when IDs are uncertain; no free-text outputs into production.
- Gate mappings: require two independent signals (lexical/structural + LLM judgment) for cross-ontology equivalence; demote to relatedTo when confidence is borderline.
- Bias checks: run stratified audits (sex, skin tone, ancestry, rare disease) on LLM-suggested terms/mappings; escalate gaps to curators with templated remediation.
- Reproducibility: pin model, prompt, temperature, retrieval index, and ontology snapshot in an audit trail; re-run spot checks each release.

### 6.2. Operational Metrics and Target Bands

We recommend reporting the following ontology-specific metrics with suggested near-term targets:

- Identifier Fidelity Rate (IFR): proportion of LLM suggestions with correct canonical IDs. Target: ≥95%.
- Axiom Consistency Violations per 1000 additions (ACV@1k): reasoner-detected contradictions post-merge. Target: ≤1.
- Curator Minutes per Accepted Edit (CMS@Edit): average human time to review and accept. Target: ≤5 min for definitions, ≤8 for mappings.
- Mapping Precision@K with curator-in-the-loop (P@K_HITL_): precision of candidate alignments at top-*K*. Target: ≥0.90 at *K* = 5 on biomedical tracks.
- Hallucinated-ID Rate (HR-ID): fraction of suggestions containing fabricated or non-resolvable IDs. Target: =0 in production.
- Run-to-Run Stability (RS@seed): agreement of accepted suggestions across re-runs with fixed seeds/snapshots. Target: ≥98%.

### 6.3. Minimal Reporting Checklist for LLM-Assisted Curation

Authors and tool builders should report (i) model name/version and decoding params; (ii) retrieval sources and index date; (iii) ontology snapshot versions; (iv) prompts/templates (redacted only if necessary); (v) curator protocol and expertise; (vi) full confusion matrices for mapping tasks; (vii) reasoner profile and rule set; and (viii) audit logs linking accepted edits to prompts/responses.

In summary, LLMs and related AI methods are poised to usher in a new era of ontology engineering—one that is faster, more scalable, and potentially more comprehensive than ever before. Yet realizing this vision will require rigorous evaluation, transparent governance, and a commitment to equity. The most promising future lies not in replacing human expertise but in human–AI collaboration, where LLMs accelerate knowledge discovery while experts safeguard accuracy, logic, and ethics. By pursuing hybrid systems, robust evaluation frameworks, collaborative platforms, bias-aware methods, continual adaptation, and clear governance structures, the biomedical community can build ontology ecosystems that are not only more efficient but also more trustworthy, inclusive, and aligned with the needs of science and healthcare.

## Figures and Tables

**Table 1:** Summary of ontology-related tasks, representative LLM-based approaches, and their strengths and weaknesses.

Task	Representative LLM Tools/Methods	Strengths	Weaknesses/Limitations
Ontology creation and enrichment	GPT-4, ChatGPT; DRAGON-AI [19]; ontology enrichment pipelines [76]	Draft new terms and definitions; accelerate ontology expansion; capture synonyms from literature	Risk of hallucinated terms or fabricated IDs; needs human validation; limited logical constraint handling
Ontology mapping and alignment	MILA [35]; GenOM (2025) [60]; embedding-based alignment methods	Leverage LLM embeddings for cross-ontology matching; capture semantic similarity beyond string overlap	Sensitive to prompt design; error-prone with rare terms; struggles with semantic disambiguation
Text mining and semantic search	GPT-3.5/GPT-4 for NER; SPIRES [18]; FuncFetch [13]	Handle biomedical synonyms; flexible extraction from unstructured text; improve recall for retrieval	Struggle with ambiguity (e.g., hypertension vs. hypotension); variable precision; ontology ID grounding remains challenging
Ontology alignment with knowledge graphs	RELATE [53]; LLM-assisted RDF generation [72]	Bridge unstructured and structured data; generate candidate triples; support semantic integration	Require symbolic reasoning validation; scalability issues; spurious relations without ontology constraints
Curation and interactive editing	Conversational editing prototypes; collaborative LLM–ontology platforms	Reduce curator workload; provide interactive drafts; support human-in-the- loop workflows	Dependence on expert oversight; reproducibility issues; lack of standardized evaluation metrics

**Table 2:** Comparison of ontology integration methods by scalability, maturity, and limitations.

Method Class	Demonstrated at Scale?	Key Strengths	Key Limitations
Rule-based/Heuristic Matchers (e.g., AML, LogMap)	Yes (production deployments)	High precision, deterministic behavior, easy reproducibility	Low recall for semantically distant terms; limited adaptability to new ontologies; minimal contextual understanding.
Hybrid LLM-Assisted Matchers (e.g., MapperGPT, MILA, SPIREX)	Emerging (pilot-scale validation)	Improved recall and contextual sensitivity; curator-in-the-loop validation; scalable through selective prompting	Requires manual oversight; lacks standardized runtime benchmarks; higher compute cost than rule-based systems.
Pure LLM-Based Systems (e.g., OntoTune, GenOM prototypes)	Limited (proof-of-concept)	Deep semantic reasoning, cross-domain generalization, ontology-grounded representation learning	Prone to hallucinations; poor reproducibility; logical constraint violations; unsuitable for regulated environments without validation layers.

## Data Availability

No new data were created or analyzed in this study.
